# Mastoidectomy and mastoid obliteration with autologous bone graft: a quality of life study

**DOI:** 10.1186/1916-0216-42-49

**Published:** 2013-09-23

**Authors:** George Kurien, Kate Greeff, Nahla Gomaa, Allan Ho

**Affiliations:** 1 Department of Surgery, University of Alberta, 1100 Youville Dr., 4016 Grey Nuns Hospital, T6L 5X8 Edmonton, AB, Canada; 2 University of Alberta, Edmonton, AB, Canada

**Keywords:** Mastoidectomy, Chronic otitis media, Obliteration, Autologous, Bone graft, Health status, Quality of life, Glasgow benefit inventory

## Abstract

**Background:**

A mastoid cavity resulting from a canal wall down mastoidectomy can result in major morbidity for patients due to chronic otorrhea and infection, difficulty with hearing aids and vertigo with temperature changes. Mastoid obliteration with reconstruction of the bony external ear canal recreates the normal anatomy to avoid such morbidity. Few have the studied the quality of life benefit that this procedure confers.

**Methods:**

This retrospective observational study was conducted to determine if mastoid obliteration with autologous cranial bone graft following mastoidectomy improves quality of life (QOL). Patients with cholesteatoma who had mastoidectomy with primary or secondary mastoid obliteration by a tertiary otologist were surveyed using the validated Glasgow Benefit Inventory (GBI), our primary outcome measure.

**Results:**

Fifty-eight patients were interviewed. Forty-six were primary obliteration after canal wall down mastoidectomy of a primary cholesteatoma. Twelve were secondary obliteration of an existing canal wall down mastoid cavity. Overall GBI scores were improved, with average scores of 22. Average general subscale scores were 23, physical health scores were 25, and social health scores were 22. The primary obliteration group had average scores of 19, general subscale scores of 20, physical health scores of 21, and social health scores of 22. Those with secondary obliteration scored higher, with average scores of 31, general subscale scores of 34, physical health scores of 39, and social health scores of 25.

**Conclusion:**

This study shows that mastoidectomy with obliteration using autologous cranial bone graft offers a significant QOL benefit. The GBI scores compare favourably with other otorhinolaryngology procedures. Secondary obliterations after revision mastoidectomy scored much higher than primary obliterations. This is currently the only QOL study comparing these two patient groups.

## Background

A canal wall-down mastoid cavity constitutes a major morbidity to patients with chronic ear disease. The consequences include susceptibility to infection with any water exposure, recurrent otorrhea, the need for frequent cleaning, difficulty with the use of conventional hearing aids and vertigo caused by warm or cold air or water exposure [1].

Mastoid obliteration with reconstruction of the bony external ear canal [2] is a procedure that is used to avoid all these complications. In 1911, Mosher introduced the concept of mastoid obliteration [3]. Since then various techniques and graft material have been described. Local fascial musculo-periosteal flaps, autologous grafts such as bone, cartilage and ceramic materials such as hydroxyapatite have been used [2,4-7].

Palva [8] introduced the use of bone chips and bone pate in combination with a musculo-periosteal flap to obliterate the mastoid cavity [8]. This has been recommended as a primary procedure at the time of canal wall down mastoidectomy. However, for a problematic non-healing mastoid cavity with chronic otorrhea, a secondary or revision procedure could be done.

Very few studies have been published regarding the quality of life (QOL) change due to primary or secondary mastoid obliteration. Dornhoffer et al. [9] studied the impact of the secondary mastoid obliteration on QOL using the Glasgow Benefit Inventory (GBI). The majority of their patients reported improved QOL and control of the otorrhea afterwards. However, to date, no comparison has been done on the impact on QOL between primary mastoid obliteration and secondary obliteration.

The GBI was first developed by Robinson at al. [10] to measure the change in health status due to an intervention. It is a validated, retrospective, single administration test which captures change in health status brought about by a specific event. The questionnaire was designed as an adult otorhinolaryngology (ORL) survey but has been used in other specialties [11,12] and also in the pediatric age group [13,14].

In otolaryngology, the GBI showed improvement in quality of life after different procedures such as intratympanic dexamethasone [15] and intratympanic gentamicin for Meniere’s Disease [16], rhinoplasty [17], endoscopic dacryocystorhinostomy (DCR) [12], and tonsillectomy [18,19]. It also showed minimal improvement of overall health-related quality of life after septoplasty [20]. In otology and neurotology, it has been used to assess QOL in acoustic neuroma patients following microsurgery [21], after bone anchored hearing aids [13,14,22] and after stapes surgery [23].

The aim of this study is to describe the change in health status in patients after mastoidectomy and mastoid obliteration. It also looks at the change in QOL between mastoidectomy with primary mastoid obliteration and secondary obliteration.

## Methods

### Patient selection

This is a retrospective QOL study. Ethics approval was obtained at University of Alberta Research Ethics Office (Pro00026634). Seventy-two patients who had undergone canal wall down mastoidectomy with mastoid obliteration using autologous cranial bone graft from 2009 till 2012 were identified as possible candidates. Patients with primary cholesteatoma who had mastoidectomy and mastoid obliteration were defined as having primary obliteration. Secondary obliterations were those with an existing canal wall down mastoidectomy who were having otorrhoea and who had revision mastoidectomy and mastoid obliteration. All patients were operated on by one tertiary otologist, therefore standardizing technique.

Twenty-four patients who had primary obliteration were contacted over the phone by the interviewer who went through the GBI with these patients. Forty-eight patients were identified as requiring an interview in person during their clinic visit. This group consisted of those who had secondary obliterations, multiple ear surgeries, and patients who had primary surgery but had a language barrier that hindered communication over the phone. Two patients out of this last group were excluded from the study due to non-compliance with postoperative treatment and follow up. In addition, one patient just had surgery recently and it was too soon to interview him. Another 12 could not be contacted either because they were out of country or had changed addresses. Ultimately, only 34 of these 48 patients completed a GBI in clinic with the help of an interviewer. 58 of the 72 patients, 81% of the originally identified patients, completed their GBI questionnaires.

### Surgical technique

In the primary procedure, a post-auricular incision was performed. A U-shaped superiorly pedicled temporalis muscle flap was raised off the mastoid bone consisting of temporalis muscle, temporalis fascia and some pericranium. The temporalis muscle was dissected from the underlying mastoid bone from the mastoid tip superiorly to the temporal line. The muscle was then retracted superiorly to expose 1 to 2 cm of the pericranium above the temporal line. Bone chips were harvested with a large chisel and mallet from the healthy, disease-free cortex of the mastoid bone before the mastoidectomy was performed so that the cranial autologous bone graft was not contaminated by disease. The bone chips were laid in cool normal saline to keep fresh. Bone pate was then collected in a special bone dust collector from the lateral mastoid cortex and laid to dry. The mastoidectomy was begun after harvesting the bone graft. At the end of the canal wall down mastoidectomy, the bone chips were used to obliterate the mastoid cavity and the epitympanum. The bone pate was used to resurface and reconstruct the external ear canal. Temporalis fascia was used to provide soft tissue cover over the bone graft onto which the remaining skin flaps of the ear canal were placed.

In the secondary procedure the only difference was that the pre-existing mastoid cavity was already present before the harvesting of the bone graft. Again, like the primary procedure the autologous cranial bone graft, in the form of bone chips and bone pate were harvested in the same way prior to breaking into the mastoid cavity. All the skin lining the mastoid cavity was removed so as to not risk burying a cholesteatoma during the mastoid obliteration. After the completion of the revision mastoidectomy, the task of obliterating the mastoid cavity with bone chips and resurfacing the bony external ear canal with bone pate progressed in just the same way as the primary procedure.

### Outcome measure

**Figure 1:**
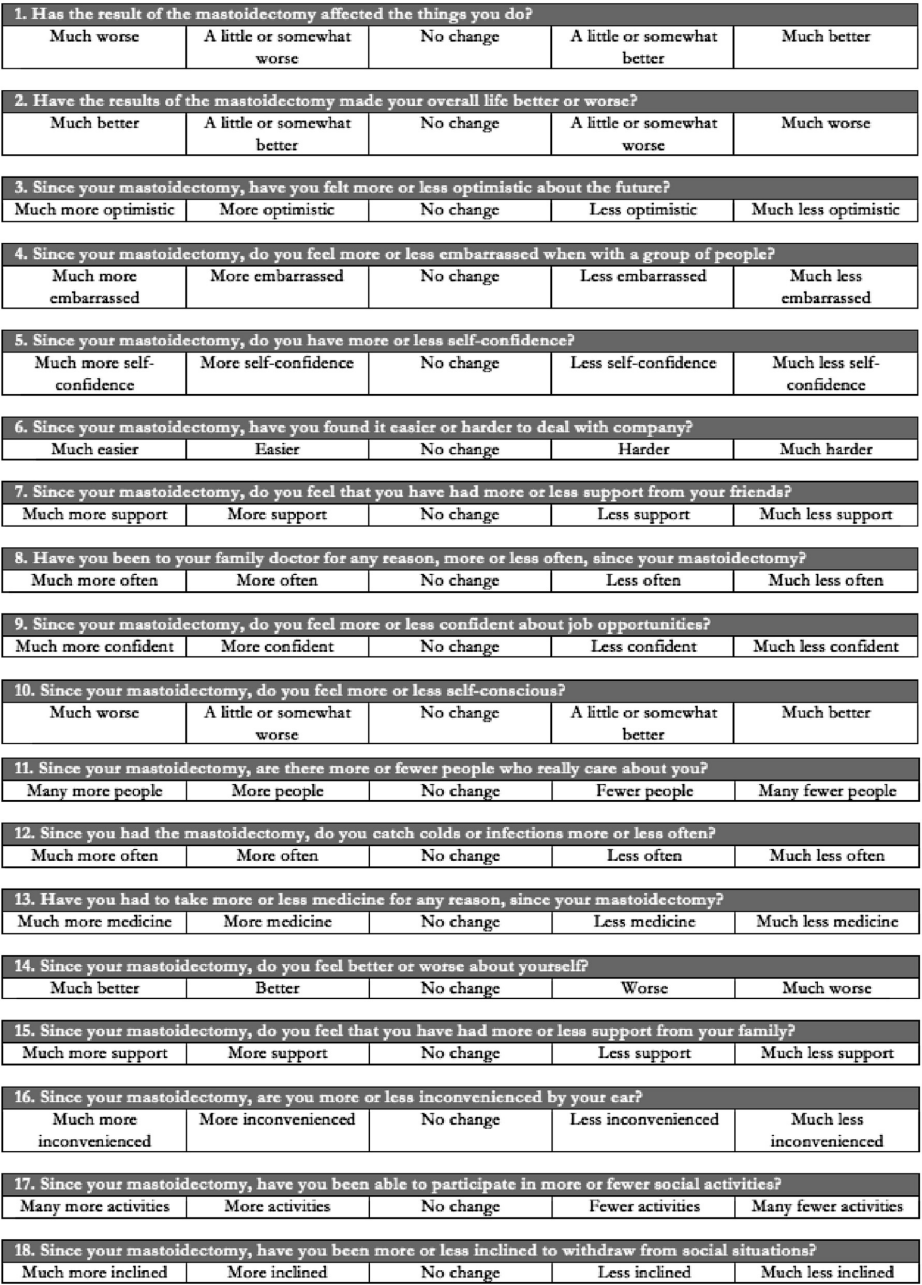
GBI questionnaire.

QOL of our 58 patients were measured using the Glasgow Benefit Inventory (GBI) survey described by Robinson et al. [10]. Figure 1 is the GBI we used. The survey consists of 18 questions. Of the eighteen GBI questions, twelve correspond to general improvement of QOL, three to social improvement, and three to physical improvement. Each question asks the patient to indicate their response on a Likert one to five scale. It was chosen as our main outcome measure because it is a validated post-intervention change in QOL questionnaire that does not require pre-intervention administration of the questionnaire. This was the ideal tool for our retrospective QOL study. As it has been used widely to survey QOL change of several otorhinolaryngology procedures, we would also be able to assess how this specific surgical procedure ranks with other otolaryngology procedures. GBI scores were calculated as per Robinson [10]. Each calculated domain had a scale from −100 to +100 with zero being no change from the intervention, -100 being maximal deterioration and +100 being maximum QOL improvement.

A chart review was done to obtain demographic and additional information such as the duration of symptoms before the obliteration was attempted, date of surgery, and whether the patient had other middle ear surgeries. Statistical analysis was performed using SPSS.

## Results

### Patient demographics

Fifty-eight patients were interviewed in person or via telephone from October 2012 to January 2013. Average patient age was forty years (Range 11–80, SD 16), and thirty-one patients were male. Twenty-eight right ears (48%) were operated on. Forty-six were primary cases (79%), while twelve (21%) were secondary, with average time to obliteration of existing cavity being 2.9 years (Range 0–42, SD 8.5) (Table 1).

**Table 1:** Patient characteristics (n = 58)

	**Total (n = 58)**	**Primary (n = 46)**	**Secondary (n = 12)**
Average age (std dev) in years	40 (16)	43 (16)	30 (14)
Male	31	24	7
Female	27	22	5
Right ears	28	24	4
Left ears	30	22	8

### Complications

Postoperative complications included five temporalis fascia graft infections that eventually achieved full epithelialization. Infection delayed full healing by approximately three weeks. There were also two ear canal irregularities requiring minor canalplasties, and one tympanic membrane perforation that eventually healed. Recurrence of cholesteatoma was encountered in four patients (6.8%), with three recurrences in the epitympanum and one in the oval window region. Recurrence was never in the obliterated mastoid cavity and no cases required the mastoid obliteration to be taken down.

### GBI scores

Evaluating our entire cohort of 58 patients, overall GBI scores were improved, with average overall scores of +22. The subgroup with primary obliteration had an average total GBI score of +19. Primary canal wall down mastoidectomy with mastoid obliteration with autologous cranial bone graft confers improvement in QOL. The subgroup with secondary obliteration of an existing cavity had an average total GBI score of +31. Comparing the primary and secondary obliteration subgroups, the difference in overall GBI score was not statistically significant (Mann Whitney U test p = 0.14). A summary of the raw GBI questionnaire data is provided in Table 2.

**Table 2:** GBI summary of results

**Question**	**Median**	**Interquartile range**	**Number of respondents per answer**				
			**5**	**4**	**3**	**2**	**1**
1. Effect on life	4	2	21	12	15	8	2
2. Overall effect on life	4	2	22	19	12	3	2
3. Optimism about future	4	2	17	23	14	3	1
4. Embarrassment	3	1	11	13	29	3	2
5. Self-confidence	3.5	1	9	20	27	1	1
6. Dealing with company	3	1	7	16	26	7	2
7. Support from friends	3	1	4	24	30	0	0
8. Visits to GP	3	1	8	16	32	1	1
9. Job opportunities	3	1	7	15	30	6	0
10. Self- consciousness	3	1	9	7	36	6	0
11. People who care	3	0	4	6	48	0	0
12. Frequency of illness	3	1	10	11	33	4	0
13. Frequency of medication	3	1	12	10	33	3	0
14. Self-opinion	4	2	16	20	17	4	1
15. Family support	3	1	8	16	34	0	0
16. Inconvenience	4	1.25	14	18	16	8	3
17. Social activities	3	1	6	13	36	3	0
18. Social situations	3	1	4	11	37	5	1

After primary obliteration, 33 of 46 (72%) reported improved QOL compared to 10 of 12 (83%) after secondary obliteration. With regard to the general, physical, and social subscales, while both primary and secondary obliteration patients reported an improved quality of life, those with secondary obliteration reported a more dramatic change in quality of life. Revision canal wall down mastoidectomy with secondary mastoid obliteration to reconstruct the bony external ear canal with autologous cranial bone graft confers great improvement of QOL. A comparison between subgroups is summarized in Table 3. The general subscale scores was +23 for the entire cohort of 58 patients, +20 for the primary obliteration group (n = 47) and +34 in the secondary obliteration group (n = 12). This difference was not statistically significant (Mann Whitney U test p = 0.10). As for the physical health subscale scores, our entire cohort scored +25 whilst the primary obliteration group scored +21 and the secondary obliteration group score +39. This difference was not statistically significant (Mann Whitney U test p = 0.15). Our entire cohort and primary obliteration group scored +22 for social support. The secondary obliteration group scored +25. This difference was not statistically significant (Mann Whitney U test p = 0.43).

**Table 3:** GBI subscale results by timing of obliteration

	**Entire cohort**	**Primary obliteration**	**Secondary obliteration**	**Mann–Whitney test (Primary vs Secondary)**
Overall (mean)	22	19	31	0.14
General subscale (mean)	23	20	34	0.10
Physical subscale (mean)	25	21	39	0.15
Social subscale (mean)	22	22	25	0.43

For the entire cohort, the most positive changes in health status were seen in change in overall effect on life (Question 2), their optimism about the future (Question 3), feeling better about themselves (Question 14), and in the things that the patient did (Question 1). For the primary obliteration group, the most positive effects were reflected in the answers from the same four questions. For the secondary obliteration group, the most positive changes in health status were seen in things that the patient did (Question 1), their optimism about the future (Question 3), feeling better about themselves (Question 14), being less inconvenienced by their ears (Question 16), and having more self-confidence (Question 5).

## Discussion

This study is the first study reporting significant improvement in QOL with mastoidectomy, mastoid obliteration and external ear canal reconstruction using autologous cranial bone graft in a Canadian population.

The GBI captured just a few QOL factors that experienced most change in health status for our cohort of patients. Questions regarding change in what things patients did, overall effect on life, optimism about the future, patients’ self-opinion were reported as clinically significant positive improvements by our entire cohort. In the secondary obliteration group positive change in self- confidence and being less inconvenienced by their ears were also reported as significant changes. Dornhoffer et al. [9] also reports positive changes in effect on life, self- confidence and being less inconvenienced. Being less self-conscious, less embarrassed and improvement in social situations were also reported by his patients but these were not found to be as significant in our patients.

Our GBI results compare favourably with the GBI scores for general otorhinolaryngology procedures such as tonsillectomy +19 [19], septoplasty +11 [20], rhinoplasty +20 [17], endoscopic sinus surgery +23 [24] and endoscopic DCR +32 [12].

Our GBI scores are consistent with the current literature reporting improved health status after surgery in patients suffering from chronic otitis media. Robinson et al. [10] reported that the total GBI score for patients who experience no discharge after chronic ear surgery was +17. Of their cases 122 had mastoid surgery and 16 had myringoplasty for otorrhoea. Bergin [25] used the GBI to evaluate the impact of different otology procedures. He found that tympano-mastoidectomy had a mean total GBI score of 3.8, mean general GBI score of 2.0. Patients who had ossiculoplasty and tympano-mastoidectomy in his study had a mean total GBI score of 14.2, mean general GBI score of 18.5. His analyses of revision mastoidectomy patients reported a mean total score of 23.9, mean general score of 22.5. Dornhoffer et al. [9] reported a mean total GBI score of +28.9 for their 23 patients who had revision mastoidectomy and secondary obliteration of their mastoid cavity using demineralised bone matrix and autologous auricular cartilage. Our cohort of secondary mastoid obliteration with autologous bone graft scored +31.

The GBI scores of our patients were similar to other otology procedures such as ossicular manipulation +16.6 [25], stapedectomy +23.3 [25], stapedotomy +29 [23], intratympanic gentamicin for Meniere’s disease +30 [16] and intratympanic dexamthasone for Meniere’s + 30 [15]. Studies on BAHA +34 [13], ossiculoplasty +34 [10] and cochlear implantation +40 [10] reported higher total GBI scores.

To date, our study is the first and largest study using a validated change in QOL tool to compare the change in QOL between primary and secondary mastoid obliteration with autologous cranial bone graft. One recent study comparing change in health status between primary and revision mastoidectomy groups [26] reported improvement in QOL in both groups 1 year after surgery. Their patients had a combination of canal wall up mastoidectomy or canal wall down mastoidectomy without mastoid obliteration. They reported that improvement in QOL was greater in the primary surgery group compared with the group who had revision mastoidectomy. Contrary to their results, our experience was that the change in QOL is more readily apparent in the patients who had secondary obliteration as opposed to primary obliteration group. This stands to reason, as the patients who had secondary obliteration without mastoid obliteration had significant “mastoid misery” and otorrhea with their canal wall down mastoid cavities. We believe that the GBI captured the true change in QOL with this group and also demonstrated the lesser clinical change in QOL in the group who had primary mastoid obliteration at the time of their first canal wall down mastoidectomy. However, this clinically noted difference was not statistically significant using the Mann–Whitney analysis when the level of significance was set at p < 0.05. The smaller number of patients in the secondary group compared with the larger primary group may be the reason for this result. We therefore suggest that further prospective study on a larger group of patients who have had secondary mastoid obliteration using this technique would be a useful addition to the current literature.

## Conclusion

Mastoidectomy with obliteration using autologous bone graft is one of the treatment options for patients with chronic otitis media. Mastoidectomy with obliteration provides a quality of life benefit to patients, and this appears to be more pronounced in those with secondary obliteration.

## Competing interests

The authors declare that they have no competing interests.

## Authors’ contributions

GK worked on literature review, data collection and analysis, and manuscript preparation. KG worked on data collection, chart review, and manuscript preparation. NG worked on literature review, data collection, chart review, and manuscript preparation. AH worked on study proposal, data collection, manuscript preparation, and coordination. All authors read and approved the final manuscript.

## References

[B1] Mehta RP Harris JP Mastoid obliteration Otolaryngol Clin North Am 2006 39 1129 1142 10.1016/j.otc.2006.08.007 17097437

[B2] Roberson JB Mason TP Stidham KR Mastoid obliteration: autogenous cranial bone pate reconstruction Otol Neurotol 2003 24 132 140 10.1097/00129492-200303000-00002 12621322

[B3] Mosher HP A method of filling the excavated mastoid with a flap from the back of the auricle Laryngolscope 1911 21 1158 1163

[B4] Dornhoffer JL Surgical modification of the difficult mastoid cavity Otolaryngol Head Neck Surg 1999 120 361 367 10.1016/S0194-5998(99)70276-7 10064639

[B5] Estrem SA Highfill G Hydroxyapatite canal wall reconstruction/mastoid obliteration Otolaryngol Head Neck Surg 1999 120 345 349 10.1016/S0194-5998(99)70273-1 10064636

[B6] Mills RP Surgical management of the discharging mastoid cavity J Laryngol Otol 1988 16 suppl 1 6 3171364

[B7] Shea MC Gardner G Jr Simpson ME Mastoid obliteration with bone Otolaryngol Clin North Am 1972 5 161 172 4551409

[B8] Palva T Mastoid obliteration Acta Otolaryngol 1979 360 suppl 152 154 10.3109/00016487809123502 377902

[B9] Dornhoffer JL Smith J Richter G Impact on quality of life after mastoid obliteration Laryngoscope 2008 118 1427 1432 10.1097/MLG.0b013e318173dd7e 18475206

[B10] Robinson K Gatehouse S Browning GG Measuring patient benefit from otorhinolaryngological surgery and therapy Ann Otol Rhinol Laryngol 1996 105 415 422 8638891 10.1177/000348949610500601

[B11] Freilich DA Penna FJ Nelson CP Parental satisfaction after open versus robot assisted laparoscopic pyeloplasty: results from modified Glasgow Children’s benefit inventory survey J Urol 2010 83 704 708 20022046 10.1016/j.juro.2009.10.040

[B12] Spielmann PM Hathorn I Ahsan F The impact of endonasal dacryocystorhinostomy (DCR), on patient health status as assessed by Glasgow benefit inventory Rhinology 2009 47 48 50 19382495

[B13] Arunachalam PS Kilby D Meeikle D Bone-anchored hearing aid quality of life assessed by Glasgow benefit inventory Laryngoscope 2001 111 1260 1263 10.1097/00005537-200107000-00022 11568551

[B14] Dutt SN McDermott AL Jelbert A The Glasgow benefit inventory in the evaluation of patient satisfaction with the bone-anchored hearing aid: quality of life issues J Laryngol Otol 2002 28 suppl 7 14 10.1258/0022215021911284 12138792

[B15] Kyrodimos E Aidonis I Skalimis A Use of Glasgow Benefit Inventory (GBI) in Meniere’s disease managed with intratympanic Dexamethasone perfusion: quality of life assessment Auris Nasus Larynx 2011 38 172 177 10.1016/j.anl.2010.07.009 20810226

[B16] Banerjee AS Johnson IJ Intratympanic gentamicin for Meniere’s disease: effect on quality of life as assessed by Glasgow benefit inventory J Laryngol Otol 2006 120 827 831 16707038 10.1017/S0022215106001605

[B17] Draper MR Salam MA Kumar S Change in health status after rhinoplasty J Otolaryngol 2007 36 13 16 10.2310/7070.2006.0050 17376345

[B18] Richards AL Bailey M Hooper R Quality of life effect of tonsillectomy in a young adult group ANZ J Surg 2007 77 988 990 10.1111/j.1445-2197.2007.04296.x 17931263

[B19] Senska G Ellerman S Ernst S Recurrent tonsillitis in adults: quality of life after tonsillectomy Dtsch Arztebl Int 2010 107 622 628 20948776 10.3238/arztebl.2010.0622 PMC2947847

[B20] Calder NJ Swan IR Outcomes of septal surgery J Laryngol Otol 2007 121 1060 1063 17349096 10.1017/S0022215107006500

[B21] Brooker JE Fletcher JM Dally MJ Quality of life among acoustic neuroma patients managed by microsurgery, radiation, or observation Otol Neurotol 2010 31 977 984 10.1097/MAO.0b013e3181e8ca55 20601919

[B22] de Wolf MJ Shival ML Hol MK Benefit and quality of life in older bone-anchored hearing aid users Otol Neurotol 2010 31 766 772 10.1097/MAO.0b013e3181e3d740 20581615

[B23] Subramaniam K Elkelboom RH Marino R Patient’s quality of life and hearing outcomes after stapes surgery Clin Otolaryngol 2006 31 273 279 10.1111/j.1749-4486.2006.01237.x 16911642

[B24] Mehanna H Mills J Kelly B Benefit from endoscopic sinus surgery Clin Otolaryngol Allied Sci 2002 27 464 471 10.1046/j.1365-2273.2002.00610.x 12472513

[B25] Bergin MJ Inner ear effects of middle ear surgery. MMSc thesis 2011 University of Otago

[B26] Jung KH Cho YS Hong SH Quality of life assessment after primary and revision ear surgery using the chronic ear survey Arch Otolaryngol Head Neck Surg 2010 136 358 365 10.1001/archoto.2010.24 20403852

